# The Dog Mite, *Demodex canis*: Prevalence, Fungal Co-Infection, Reactions to Light, and Hair Follicle Apoptosis

**DOI:** 10.1673/031.011.7601

**Published:** 2011-06-29

**Authors:** Yu-Jen Tsai, Wen-Cheng Chung, Lian-Chen Wang, Yu-Ten Ju, Chin-Lin Hong, Yu-Yang Tsai, Yi-Hung Li, Ying-Ling Wu

**Affiliations:** ^1^ Taipei City Animal Protection Office, No. 109, Ln. 600, Wu-Xin Street, Taipei 11048, Taiwan; ^2^ Department of Parasitology, Taipei Medical University, No. 250, Wu-Xin Street,Taipei 11042, Taiwan; ^3^ Graduate Institute of Biomedical Sciences, Chang Gung University, College of Medicine, 259 Wen-Hwa 1st Road, Kwei-Shan Tao-Yuan 33302, Taiwan; ^4^ Department of Animal Science and Technology, National Taiwan University, No. 1, Sec. 4, Roosevelt Road, Taipei 10617, Taiwan; ^5^ Department of Public Health, Taipei Medical University, No. 250, Wu-Xin Street, Taipei 11042, Taiwan; ^6^ Land Environmental Information Consulting Association, 20F-1, No.6 Bau Ching St. Hsing-dian, Taipei 23143, Taiwan; ^7^ School of Veterinary Medicine National Taiwan University, No. 1, Sec. 4, Roosevelt Road, Taipei 10617, Taiwan

**Keywords:** fungi, map overlay, Taiwan

## Abstract

Infection rate, reaction to light, and hair follicle apoptosis are examined in the dogmite, *Demodex canis* Leydig (Prostigmata: Demodicidae), in dogs from the northern area of Taiwan. An analysis of relevant samples revealed 7.2% (73/1013) prevalence of *D. canis* infection. Infection during the investigation peaked each winter, with an average prevalence of 12.5% (32/255). The infection rates significantly varied in accordance with month, sex, age, and breed (p < 0.05). Most of the lesions were discovered on the backs of the infected animals, where the infection rate was 52.1% (38/73) (P < 0.05). The epidemiologic analysis of infection based on landscape area factor, found that employing a map-overlapping method showed a higher infection rate in the eastern distribution of Taiwan's northern area than other areas. Isolation tests for *Microsporum canis* Bodin (Onygenales: Arthrodermataceae) and *Trichophyton mentagrophyte* Robin (Blanchard) on the *D. canis* infected dogs revealed prevalence rates of 4.4% (2/45) and 2.2% (1/45), respectively. Observations demonstrated that *D. canis* slowly moved from a light area to a dark area. Skin samples were examined for cellular apoptosis by activated caspase3 immunohistochemical staining. Cells that surrounded the infected hair follicles were activated caspase3-positive, revealing cell apoptosis in infected follicles via the activation of caspase3.

## Introduction

*Demodex canis* Leydig (Prostigmata: Demodicidae) is an ectoparasite which lives in the hair follicles and sebaceous glands of various animals. Its primary food source is from the secretions of follicular glands or sebaceous glands. Under normal conditions, it does not cause skin disorders. However, *D. folliculorum* increases in number (Lee 1987; Hsu et al. 1992; Desch and Hillier 2003) when immunity is weakened or suppressed. Apart from size differences it is hard to distinguish different species. Most species are named according to the hosts that they parasitize, e.g. dogs (*D. canis*), sheep (*D. ovis*), goats (*D. caprae*), cattle (*D. bovis*), pigs (*D. phylloides.*), hamsters (*D. criceti*), and cats (*D. cati* and another species yet unnamed) (Lee 1987; Wu 1989; Xia and Hao 2003). Mimioglu et al. (1979) and Morsy et al. (1995) have pointed out that both humans and dogs may be infected with *D. folliculorum* (Mimioglu et al. 1979; Morsy et al. 1995). Most studies of *Demodex* in dogs have been independent of each other, and no systematic or regional studies of this parasite appear to have been conducted. The aim of this investigation is to establish a local database of *D. canis* in Taiwan.

## Materials and Methods

### Area of investigation and samples

The area of study included 12 administrative districts of Taipei (121° 27′ ∼ 121° 40′ N and 25° 13′∼24° 08′ E) and 1013 canine skin samples (187 pet and 826 stray dogs) were examined. Skin scrapings from head, body, and four limbs were collected using sterile scalpel blades and applied to glass slides. The skin scrapings were covered with a solution of clearing reagent (10% KOH) for 3–5 minutes before microscopic examination. Selected dogs were also tested by an adhesive tape test, and some selected dogs with *D. canis* infection had skin scrapings cultured on a selective medium for isolating fungi as described below.

### Microscopic examination

The standard procedure for microscopic detection of *D. canis* has been described previously (Chang and Yueh 1983; Yu and Xu 1992).

### Adhesive tape test

Thirty randomly selected dogs (15 males, 15 females) were selected from 73 dogs that been confirmed to be infected with *D. canis,* and were examined by the adhesive tape method described by Wu et al. (1992). Transparent adhesive tape (310 Scotch, M www.3m.com) was applied to a shaved area of skin in the morning (08:00–10:00) and at night (20:00– 22:00) (periocular area, back and forelimbs). The adhesive tape was examined microscopically and infection was classified as minor, medium, or severe based on the number of *D. canis* detected microscopically — 1–9, 10–19, and ≥ 20, respectively. Environmental sampling tests (at 323 places on dog cages and the floors where the canines were kept during the study) were also performed using the adhesive tape test.

### Reaction to light

*D. canis* reaction to light is briefly described. A combination of the Yiwada method and the formalin-ether concentration sedimentation method was applied to test the samples (Chang 1980). The method involved adding 100c.c. saline to the sample and stirring the mixture thoroughly using a glass rod, before the mixture was filtered through a metal screen (150 mesh), and poured into a centrifugal tube to undergo separation at 447 × g, for three minutes. The upper suspension was removed until approximately 5 ml of residue was obtained; 1 ml of saline water was then added and the centrifuged at 447 × g for 3 minutes. The process was repeated three times and then one drop of the mite-containing liquid was placed on the prepared cavity slide (on the underside of which was attached black opaque tape stuck in which is cut a hole with a diameter of 2 mm diameter to let light through). The sample was then examined in a darkroom.

### Isolation of fungi

Forty-five (25 males, 20 females) of the 73 dogs which had already been confirmed to have been infected with *D. canis* were examined for presence of dermatophyte fungi in the *D. canis* positive skin samples by cultivation using Sabouraud's dextrose agar (Van Cutsem et al. 1985a; Kao 1998; Scott et al. 2001).

### Counting eosinophil in peripheral blood

Thirty of the dogs that were confirmed to have been infected with *D. folliculum* were randomly selected, and their blood was examined (Liu's stain) for the presence of eosinophilia (Chang 1980).

### Pathogen and apoptosis

Five of the 73 dogs that had been infected with *D. canis,* were randomly selected. Full-thickness skin samples were obtained from the skin lesions for histopathological and immunohistochemical examinations. Tissue slides were fixed in 4% paraformaldehyde, and 15 µm-thick cryosections were obtained. These cryosections were permeabilized with 0.05% Triton X-100, and then stained with a specific anti-activated caspase3 antibody (Chemicon Inc., www.chemicon.com). The sections were then incubated with horseradish peroxidase-conjugated anti-rabbit IgG antibody (1: 300, Amersham Biosciences, www.apbiotech.com) for 2 h at room temperature. The peroxidase-labeled secondary antibody was detected by diaminobenzidine tetrahydrochloride precipitation using an ABC kit (Vectastain Elite, www.vectorlabs.com) following the manufacturer's instructions. To determine whether hair follicle cells from follicles infected by *D. canis* showed evidence of apoptosis, five cryosections from infected skins and sections from normal skins were obtained and stained (Hoechst 33342 s*tain*) to observe the staining pattern of cellular nuclear DNA for evidence of apoptosis. To determine whether the apoptotic cells were induced by activating the caspase 3 cascade, an immunohistochemical stain based on an antibody specific for activated (truncated) caspase 3 was employed to examine the skin samples.

### Map overlapping

A geographic information system (ESRI ArcView3.3, www.esri.com) was applied to overlay maps in order to elucidate the relationships between the infection of *D. canis* and the population density, the distribution of stray dogs, and the distribution of the market.

### Environmental data collection

Environmental data was drawn from the Central Weather Bureau (temperature, rainfall, relative humidity, number of rainy days, and hours of sunshine); the Department of Market Administration, Taipei City Government (statistics of distribution and numbers of the market types); the Statistical Databank of the Office of Statistics of the Department of Civil Affairs, Ministry of the Interior (population density, rivers).

### Statistical analysis

Statistical analysis was carried out using SPSS Base (version 14.0, 2007; www.spss.com) statistical analysis software and a Chi-square test (c^2^). The binominal distribution method was employed to analyze the relationship between the infection rate of *D. canis* and the gender, age, source (pet dogs or stray dogs), and breed (mixed or pure) of the dogs as well as spatial-temporal and climatic factors. α=0.05 was chosen as the standard for significance of correlation or difference (Chen 1993).

## Results

### Infection results of area, month, and positive rate

Seventy-three out of the 1013 stray dogs were infected with of *D. canis,* yielding an infection rate of 7.2% (73/1013), as shown in Table 2 and 3 .

### Adhesive tape test

The infection rates of *D. canis* in three body area samples taken in the morning (08:00∼10:00) were 30.0% (9/30), 50.0% (15/30), and 26.7% (8/30), respectively; in the evening group (20:00∼22:00) the rates were 36.7% (11/30), 73.3% (22/30), and 30.0% (9/30), respectively. The environmental *D. canis* detection rate in the cages and other locations where the dogs were kept was approximately 0.6% (3/323).

### Reaction to light

Microscopic observations demonstrated that 15 of 16 *D. canis* slowly moved from light areas to dark areas.

### Isolation of fungi

Two species of fungi, *Microsporum canis* Bodin (Onygenales: Arthrodermataceae) and *Trichophyton* mentagrophyte Robin (Blanchard), were isolated from the skin samples examined in dogs with *D. canis* infection. The infection rates for these dermatophyte fungi were 4.4% (2/45) and 2.2% (1/45), respectively, as shown in Table 1.

### Haematology examination for eosinophilia

Infection with *D. canis* did not significantly affect the number of eosinophils in the peripheral blood (p > 0.05). This showed that dogs with *D. canis* fell within the normal range for blood eosinophil concentration.

### Histopathological changes in dogs infected with *D. canis*

Under low-power (×40) microscopic examination, the epidermis was irregularly thickened by mild hyperkeratosis. Hair follicles were irregularly enlarged, and the cigar-shaped *D. canis* mites were observed in the enlarged cavity, accompanied by hyperplasia of the keratinocytes. Mononuclear inflammatory cells including plasma cells and lymphocytes had infiltrated around the enlarged hair follicles.

### Shrinkage of hair follicles in *D. canis-*infected skin induced apoptosis by activating caspase 3

**Figure 1.**
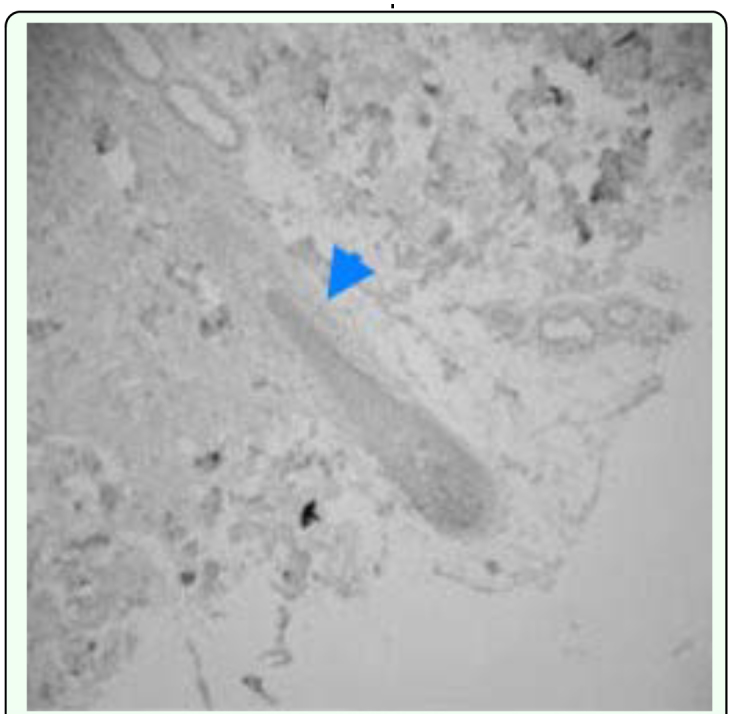
This hair follicle vessel shows the activation of caspase3 (arrow) of *Demodex canis* (experiment). High quality figures are available online.

The nuclei of *D.* canis-infected follicle cells revealed a high proportion of fragmented DNA compared with follicle cells from normal skin, indicating that these cells underwent apoptosis following *Demodex folliculorum* infection. The infected hair follicle cells were positive for activated caspase 3, confirming the activation of this enzyme (Figure 1), which results in apoptosis of the cells and the eventual loss of hair from affected follicles.

### Map Overlapping

GIS spatial analysis demonstrated no relationship between the administrative area or market type (traditional, wholesale, or concentrated) and the rate of infection by *D. canis.* (Tables 1 and 5). Climactic factors (average temperature, average rainfall, average relative humidity, number of rainy days, hours of sunshine, total amount of ozone) were correlated with the infection rate of *D. canis* (p > 0.05) (Table 4).

## Discussion

The positive rate of infection by *D. canis* in this investigation was 7.2% (73/1013), which was slightly higher than the positive rate of infection 6.5% (21/324) in another study in 2002 (Tsai et al. 2004). The infection rate in the latter investigation was within the range of that in the current study, however, the overall detected infection rate was higher than the 1.3% (2/150) as found in the investigation of infection in the Taipei area (Huang 1995). This was lower than data presented elsewhere such as 12% from Yumane (Chang and Yueh 1983), 9.2% from Kao (Kao 1999), and 23.0% from R.I. Rodriguez-Vivas (Mimioglu et al. 1979). In the current study of *D. canis* from 2003 to 2007, the observed infection rates were 6.1% (15/246), 8.5% (18/2120), 7.6% (14/184), 9.0% (16/177), and 5.2% (10/194) in 2003, 2004, 2005, 2006, and 2007, respectively. The infection rate thus ranged between 5% and 9%. The difference between the highest and lowest infection rates is approximately a 3.8% difference, which is explained by the decrease in the number of stray dogs from 7000 dogs in 2006 to 3500 dogs in 2007.

**Figure 2.**
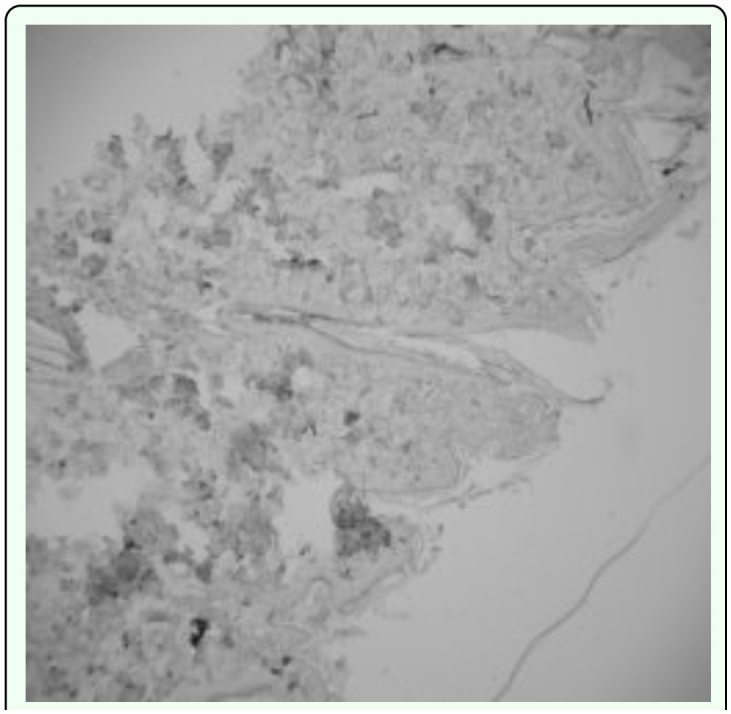
Photomicrograph showing cellular immunity stain and inactivation of caspase3 in normal skin (control). High quality figures are available online.

**Figure 3.**
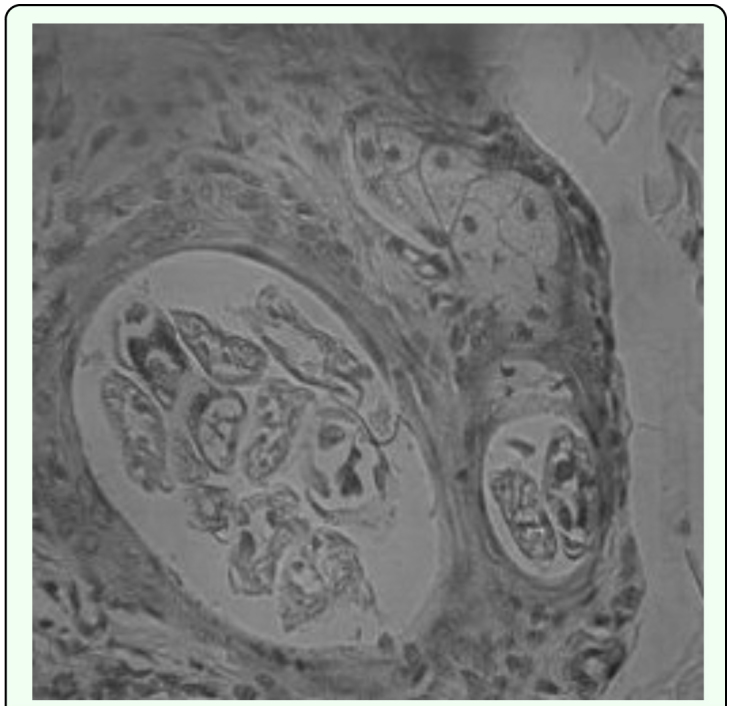
*Demodex canis* infection shown by Hoechst staining. High quality figures are available online.

The infection rates during this five year period included three peaks in February, June, and November. November 2004 had the highest rate of infection at 33.3% (3/9). No infection was observed in April or August (2003–2007) where the result was similar to that obtained in 2002 (Tsai et al. 2004). However, based on an analysis of the total sample of 1013 dogs using a c^2^ test, Neihu exhibited the highest regional positive rate of 19.2% (14/73) and June produced the highest monthly rate of 28.8% (21/73). These results are consistent with the results in 2002 (Tsai et al. 2004). The months with the highest infection rates from 2003–2007 were June of 2003 with 41.7% (5/12), November of 2004 with 33.3% (3/9), June of 2005 with 27.8% (5/18), November of 2006 with 22.1% (4/19), and June of 2007 with 15% (3/20). In summary, June is the time of year in which 60% of all of the infections were recorded during the five-year period. The infection rates varied significantly with the month (p < 0.05), showing results that are different from results in 2002 (Tsai et al. 2004).

Table 2 reports the infection rate of male dogs and female dogs by *D. canis* (p < 0.05). There is a difference related to the influences on the secretion of the sebaceous glands by male hormones and the adrenocortical hormone (Miu 1974), and the difference is consistent with the results of an earlier study by the author in 2002 in which all infection rates in male dogs exceeded those in female dogs. Pet dogs and stray dogs showed infection rates of 4.3% (8/187) and 7.9% (65/826), respectively; they did not differ significantly (p > 0.05). Mixed breeds and pure breeds exhibited rates of 6.4% (58/918) and 14.7% (14/95), respectively; these were significantly different (p < 0.05). The infection rates of dogs younger and older than two years old were 13.0% (28/216) and 5.6% (45/797), respectively; these results showed significant differences (p < 0.05). Although the two rates appear to differ by a factor of 2.3, this result is inconsistent with the results of a study of one-year-old dogs in 2002 (Tsai 1995) that found an opposite relationship, but are consistent with the study by Scott.et al. (2001).

An analysis that assumed a binomial distribution demonstrated that the part of the body with the highest infection rate of 52.1% (38/73) (p < 0.05) was the back; these results are consistent with data obtained in 2002 (Tsai et al. 2004). However, a comparison of three body areas (head, body, and limbs) of the dogs demonstrated that the body presented the highest infection rate of 93.2% (68/73). When the three areas were subdivided into smaller regions, the periocular area had the highest rate of infection, 28.8% (21/73), of any part of the head while the back of the trunk had the highest rate of infection, 52.1% (38/73), of any part of the body, and the forelegs had the highest rate of infection, 12.3% (9/73), of either pair of limbs (p > 0.05). Chen (1997) found that the back of a dog at 2.5–5 cm around from the root of the tail had more sebaceous glands than any other part of the skin, and he hypothesized that this was responsible for increased *D. canis* infection rate in that area. However, the experiment described above demonstrated that higher concentration of sebaceous glands does not necessarily correspond to a higher infection rate. Chen (1997) pointed out that younger dogs produce fewer secretions from their sebaceous glands, leading to a lower infection rate. Nonetheless, in the current experiment, dogs younger than two-years-old have a rather high infection rate, which is closer to the findings of Scott et al. (2001) who claimed that the infection rate was higher from 3 to 18 months than at older ages. However, this differs from the finding that infection rates in humans increase with age (Li and Lai 1992).

The prevalence of dermatitis in and around the ear in this study was similar to that in 2002 (Tsai et al. 2004). These results (infection rates on other parts: calvaria, mouth, periocular area, abdomen, neck, tail, anus, forelimb, and hind limb) do not indicate that mites are present in the part that presents dermatitis. The results are similar to those of Kao (1998). Hsu pointed out that hairless mice that were fed with human cortisone every day suffered from epidermal proliferation in the skin, which affected the function of the skin barrier and the skin structure (Hsu 2006). The epidermal barrier function of the area of the skin from which dermatitis samples were collected may have been affected by other causes than *D. canis* infection including primary infections (scabies, heredity or food allergy, contact allergy, seborrhoea, auto-immune disorders, infantile Pyodermia) or by secondary bacterial or fungal infections. Microbial examinations of the dermatitic ears of dogs show that 1.7– 47.6% of dogs have *Staphylococcus* spp. or fungal infections causing the exudation of serous fluid onto the affected skin (Liu et al. 1994; Morsy et al. 1995).

The part played by co-infections with dermatophyte fungi in skin of dogs with confirmed *D. canis* infections was investigated. As shown in Table 1, the dogs on which *M. canis* was detected were all younger than two years of age. The low prevalence of dermatophyte co-infection (4.4% *M. canis* and 2.2% *T. mentagraphites*) in the *D. canis* cases was similar to previous investigations (Aho 1980; Kao 1998); and was consistent with that presented by Foil, who found that *M. canis, T. mentagrophyte*, and *M. gypseum* are the most frequently detected dermatophyte fungi in dogs. The corresponding infection rates of this study are lower those found in the investigations of Kao (1998) [*M. canis,* 21.4% (83/387); *T mentagrophyte*: 16.8% (65/387)] and Tsai et al. (2004) [*M.* *canis*: 42.8% (9/21); *T. mentagrophyte*: 9.5% (2/21)], whereas the infection rate by *T. mentagrophyte* is close to that in the investigation by Brilhante et al. (2003). The infection rate of *T. mentagrophyle* in the present investigation is consistent with that obtained by Kao (1998), who found that the prevalence fluctuated seasonally but was always highest when there were high temperatures in the low-humidity season. However, the rate of co-infection of dermatophyte fungi and *D. canis* is higher in the present study than was found by Kao (1998) [1.03% (4/378)]. According to the results of Table 1, which are consistent with the results presented by Aho (1980) and Sparkes (1993), positive isolation rate for males were not found to exceed that for females. Kao (1998) found that the prevalence of Mycotic dermatitis in dogs in the Taipei metropolis was 41.9% (183/437), which significantly exceeds that in Belgium (18.3%) (Van Cutsem et al. 1985a), Finland (3.9%) (Aho 1980), and Britain (8%, Wright 1989; 9.6%, Sparkes et al 1993), but does not differ significantly from that in India (42.2%) or Italy (42%) (Ranganathan et al. 1998).

Based on topography and climactic factors [temperatur, rainfall, relative humidity, number of rainy days and hours of sun], Pearson's correlation analysis indicates significant variation of climatic factors (p < 0.05) between hours of sunshine and the year, hours of sunshine and the number of rainy days, number of rainy days and rainfall. However, the rate of infection by *D. canis* did not vary significantly with climatic factors, (p > 0.05) This result somewhat differs from previous findings in 2002 (Tsai et al. 2004). The analysis of the infection rate of *D. canis* and the distribution of households that raise dogs as well as the population density along three riversides (Keelung River, Tanshui River and Hsintien River) show no correlation. However, an analysis conducted using the map overlaying method, that mapped the distribution of stray dogs (as revealed in the stray dog distribution density map) (Neihu, Shilin, Wenshan, and Daan) and infection rate of in the east (Neihu, NanGang, Songshan, and Xinyi) of the centralized area, revealed an intersection in the Neihu district where many stray dogs are present. However, the analysis of the infection rate of *D. canis* in the 12 administrative districts considered in the present study revealed a significant difference (p < 0.05). In the southern and northern areas of the region of interest (Beitou District and Wenshan District), the observed infection rate exceeded 9% and was not correlated with human population density.

The best time to detect *D. canis,* as mentioned by Miu (1974), is at night. Hours of sunshine can influence the gender of the mites born, and the activity of the mites is related to the photoperiod and the temperature. Light and temperature affect the secretion of hormones, which indirectly establishes day-time periodicity (Miu 1974). This is consistent with the higher night-time infection rate of *Demodex spp.* detected (Miu 1974; Wu et al. 1992). Similarly, different infection rates had been obtained by sampling during different periods of sunshine and temperature in the present study. Additionally, the use of 3M transparent tape in the adhesive tape test, which was performed on 30 dogs, demonstrated that the back of the dogs had the highest rate of infection (as shown in Table 2) at night (8:00–10:00, 15/30 (50.0%) compared to 20:00–22:00 22/30 (73.3%)). The rates of infection on the periocular area, back, and forelimbs obtained during 20:00–22:00 also exceeded those during 8:00–10:00. These results were similar to those presented in Wu's study on humans (Wu et al. 1992), and show the periodicity of the activities of *D. canis.* This study has also demonstrated negative phototaxis for *D. canis in vitro.* This finding, along with other observations about negative phototaxis, suggests a possible method for improving treatment of the disease by covering the lesions with black cloth to simulate a dark environment.

In this study, the infected dogs exhibited no increase in circulating eosinophils or in lesions on histophatological examination. Activated caspase 3 immunohistochemical staining of lesions indicated that the many cells that surrounded infected hair follicles were in a caspase3-activated state, which is a precursor stage to cell apoptosis, that would in turn lead to shrinkage and loss of hair follicles in the later stages of the infection. This is consistent with reported findings on the pathogenesis of *D. canis* lesions. Immune responses in *D. canis* infections often transformed to a Th1 type response that reduces the number of eosinophils, possibly under the influence of pro-inflammatory cytokine IL2-12. In studies by Chang (2003) on the growth of hair, some hair follicle cells undergo apoptosis as part of the normal hair growth cycle. However, *Demodex-*infected skin lesions have a larger proportion of hair follicle basal cells that undergo apoptosis than skin samples from normal dogs. Other studies and the present studies have found that a large amount of kerationocyte hyperplasia that was present in hair follicle cavities in *Demodex-*infected skin. Studies by Li et al. (2001) have found that the protein caveolin secreted by caveolin cells increases in the cytoplasm of keratinized cells. In this study the large increase in keratinocyte hyperplasia was correlated with the increase of caspase 3 activation in hair follicle cells. Other studies by Nagajyothi et al. (2006) showed that upon infection by a parasite, the amount of caveolin-1 decreases affected the functions of the receptor. Galbiati et al. (2001) also observed that caveolin-1 can cause the cell to stay in the G0/G1 stage. Further studies in this area will undoubtedly lead to better understanding of the pathogenesis of *Demodex* infections.

**Table 1.**
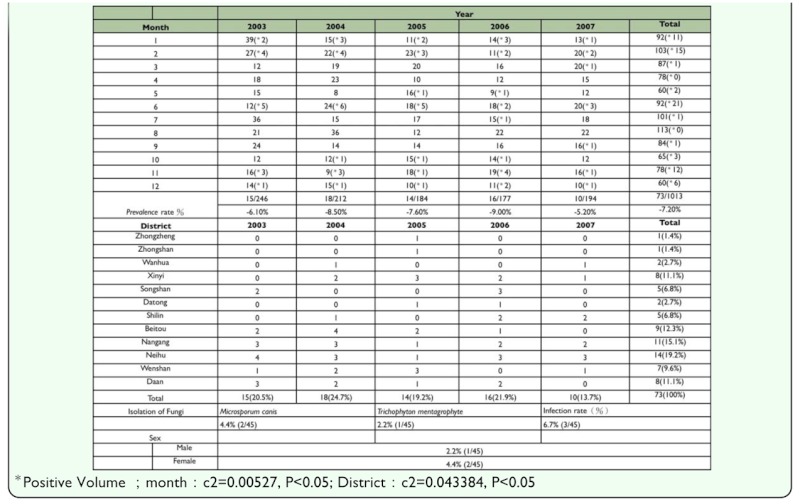
Prevalence of *Demodex canis* on stray dogs in Taipei city by month and area from January, 2003 to December, 2007.

**Table 2.**
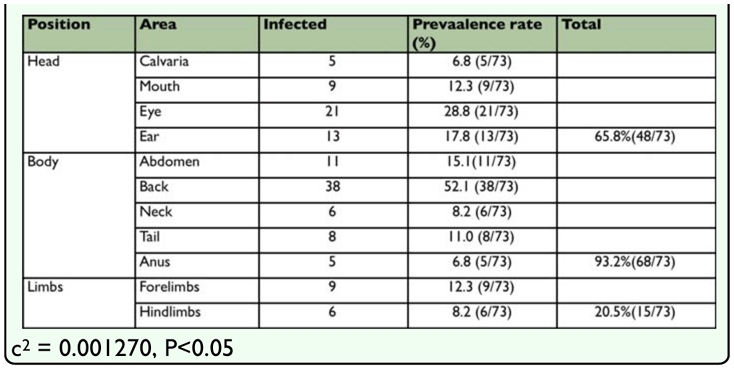
Distribution statistics of infection area of *Demodex cani* on stray dogs in Taipei city (2003–2007).

**Table 3.**
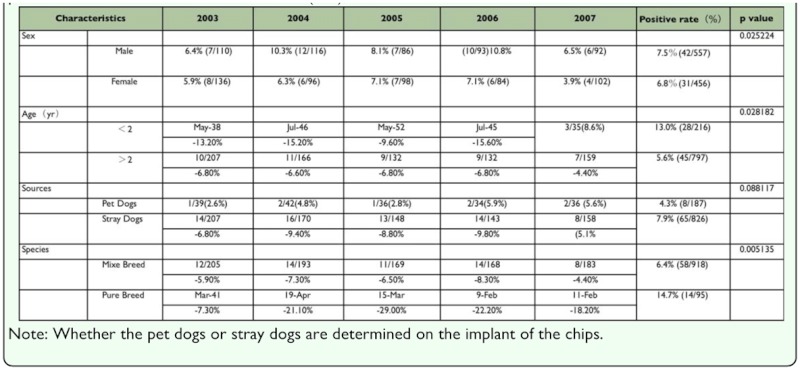
Mean (± SE) number of adelgid eggs consumed by adult *Sasajiscymnus tsugae* in a 72-hour feeding rate paired-choice test conducted at 26°C, 16:8 (LD), and 70–80 *%* RH.

**Table 4.**
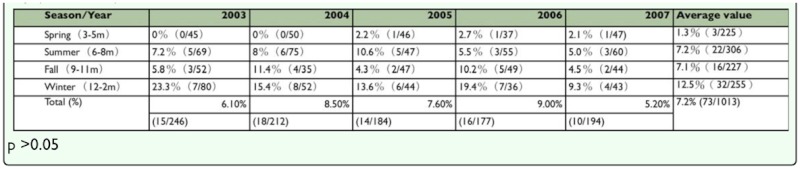
Prevalence of Demodex *canis* and analysis of landscape, climate and season factor of stray dogs in Taipei city (2003–2007).

**Table 5.**
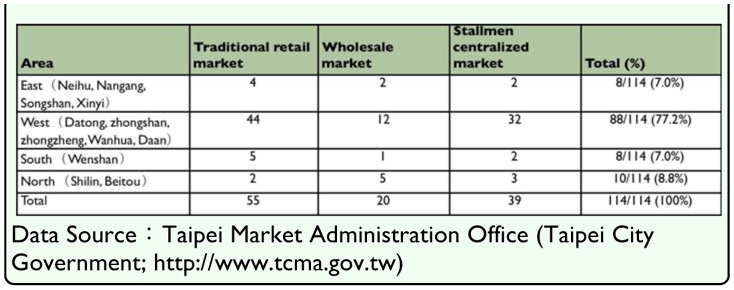
The statistic of distribution and numbers of the market types (Traditional retail, Wholesale, and Stallmen centralized) of Taipei city.
